# Urothelial carcinoma in COVID-19: lessons from a pandemic and their impact on clinical practice

**DOI:** 10.2217/fon-2021-0895

**Published:** 2021-10-21

**Authors:** Georges Abi Tayeh, Nour Khalil, Marwan Alkassis, Fouad Aoun, Chady Waked, Elie Nemr

**Affiliations:** ^1^Department of Urology, Hotel Dieu de France Hospital, Beirut, Lebanon

**Keywords:** COVID-19, diagnostic cystoscopy, neoadjuvant chemotherapy, radical cystectomy, urothelial cancer

## Abstract

The COVID-19 pandemic has modified the management of urothelial carcinoma (UC). Eighteen months after the onset of the pandemic, a scoping narrative review was able to state that radical cystectomy for UC should not be delayed beyond 10 weeks when neoadjuvant chemotherapy (NAC) was administered and 12 weeks when it was not. NAC should be considered when imminent chemotherapy cannot be performed. Early cystectomy should not be delayed when indicated for patients with high-risk non-MIBC. Patients with non-MIBC should still receive their induction doses of intravesical instillations. Diagnostic cystoscopy should not be deferred in symptomatic patients. Surgical management of upper tract urothelial carcinoma (UTUC) allows for a wider deferral interval.

The coronavirus disease 2019 (COVID-19) pandemic, caused by the SARS-CoV-2 virus, has been ongoing for around 18 months, inflicting tremendous morbidity and mortality on a global scale. Besides the well-known pulmonary and systemic complications attributed to the infection, the considerable mortality rates have called for measures aimed at slowing the spread and the eventual lethality of SARS-CoV-2 infection [1]. These measures include worldwide prolonged and stringent lockdowns and public health campaigns; but, most of all, there has been a drastic change in the operational aspects of healthcare facilities. Accordingly, most elective diagnostic or interventional procedures have been deferred, often with no preplanned patient or pathology-related strategies, and healthcare workers have been recruited from their regular specialized medical field toward COVID-oriented medical practice [1,2]. The COVID-19 pandemic has impacted urology, with lessons learned over the course of the pandemic that should prompt, when possible, appropriate patient and pathology-related guidelines for the current and future pandemics. This specific issue has been addressed since the first months of the pandemic when scientific committees put lots of effort into adapting the established guidelines to the unique character of the global pandemic to protect both physicians and patients [3]. A narrative review of the literature was performed to provide a summary of evidence regarding the most appropriate management of urothelial carcinoma (UC) of the bladder and upper urinary tract during the COVID-19 pandemic. Patients with cancer should be considered a very high-risk population for COVID-19 infection due to the neoplasm itself, its treatments and particular care sometimes requiring immunosuppressive regimens and recurrent hospital visits. Therefore, this category of patients should be of top priority for COVID-19 vaccination. The latter step may provide an additional barrier to COVID-19 infection and prevent morbidity and mortality [4,5].

In urological oncology, the adjournment of surgical interventions due to the pandemic has refueled the debate regarding the acceptable delay for muscle-invasive bladder cancer (MIBC) treatment. Boeri and Sanchez-Ortiz have advocated for an extirpative vesical intervention within 10 weeks of the last neoadjuvant chemotherapy (NAC) cycle and within 12 weeks when no NAC has been administrated. Delays prove to be deleterious regardless of age, gender or pathologic stage [6,7]. Therefore, radical cystectomy should be considered a deferred emergency rather than an elective routine procedure. However, with strict lockdown measures preventing prompt surgical interventions, NAC (when applicable) allows “buying time” until more favorable conditions permit adequate surgical treatment [8–11]. Moreover, the presence of variant histology in diagnostic transurethral resection of bladder tumor (TURB-T) should be a criterion to consider even more urgently the radical cystectomy [12]

In the setting of non-MIBC (NMIBC), guidelines were initially inspired by the fear of getting COVID-19 rather than based on a thorough risk assessment study comparing the morbidity and mortality associated with urothelial carcinoma to those infected by COVID-19 [13]. With refinement of the guidelines, it became evident that the impact of surgical delay was as important in MIBC when early cystectomy for high-risk NMIBC was compared with delayed cystectomy [14]. In the setting of high-risk NMIBC, tumor biology appears to have a stronger predictive prognostic value than the timing of surgical intervention. Concerning low-grade NMIBC, there is robust data to support the safety of deferral of cystoscopic surveillance and TURB-T for patients with known low-grade recurrences, unless new symptoms occur. As for intravesical instillations in NMIBC, there is a lack of solid data on the effect of induction delays. Therefore, these patients should receive their induction doses as soon as possible, contrary to the initial British guidelines advocating for a deferral of intravesical instillations due to fear of immunosuppression induced by Bacillus Calmette-Guérin intravesical instillations (BCG) [13]. However, maintenance therapy beyond three months may be omitted until the risk of COVID-19 infection is diminished [15,16]. If patients undergoing intravesical BCG therapy are found to be infected by COVID-19, there is evidence supporting interruption of instillations for three weeks, allowing for a full recovery, followed by treatment completion for six weeks, with a total duration of therapy not exceeding one year [17].

More importantly, strict stay-at-home orders and the postponement of elective diagnostic procedures such as cystoscopy have led to a delay in the diagnosis of bladder cancer. Delays exceeding 14 days between symptom onset and diagnosis have shown a negative survival impact, as symptomatic bladder cancer was found to correlate with a higher tumor stage [15,18]. Thus, healthcare authorities should propose virtual substitutes when closing down medical facilities (mainly telemedicine). Such closings incite patients to cope with their symptoms rather than contact a healthcare professional. Virtual ways to consult would allow determination of an appropriate strategy to evaluate symptoms while ensuring minimal physical crowding of outpatient departments [1].

Concerning upper tract urothelial carcinoma (UTUC), focused studies have demonstrated that rescheduling of treatment led to worse prognostic outcomes as a whole and specifically worse pathologic staging, presence of carcinoma *in situ* (CIS), tumoral infiltration, and so on. Determining the timing of the intervention should be done according to the UTUC risk profile. For instance, ureteral tumors have a worse prognosis than their renal pelvic counterparts when surgery is delayed beyond one month. However, patients with pT2 or greater UTUC did not show worse survival outcomes when their surgery was delayed beyond three months [19,20]. The same conclusions were drawn for metastasis-free and recurrence-free survival [21].

The COVID-19 pandemic should not prevent diagnostic evaluation of bladder cancer-related symptoms. Moreover, radical cystectomy should be preplanned to occur within 10–12 weeks from diagnosis/NAC. When possible, NAC should be considered, as it allows for a survival benefit while waiting for the proper time to perform an extirpative oncologic procedure. There is also enough evidence that UTUC allows for a wider deferral interval than bladder UC, which may prove to be vital in the setting of a global lockdown or suspension of elective operative activities. Most importantly, patients with UC should be enrolled in vaccination programs worldwide to provide them with adequate protection from COVID-19 infection, morbidity and mortality. This strategy also allows patients to attend their scheduled appointments for endoscopic follow-up and physician visits and to remain on their treatment schedules.
